# Chinese-Named Entity Recognition From Adverse Drug Event Records: Radical Embedding-Combined Dynamic Embedding–Based BERT in a Bidirectional Long Short-term Conditional Random Field (Bi-LSTM-CRF) Model

**DOI:** 10.2196/26407

**Published:** 2021-12-01

**Authors:** Hong Wu, Jiatong Ji, Haimei Tian, Yao Chen, Weihong Ge, Haixia Zhang, Feng Yu, Jianjun Zou, Mitsuhiro Nakamura, Jun Liao

**Affiliations:** 1 School of Science China Pharmaceutical University Nanjing China; 2 School of Basic Medicine and Clinical Pharmacy China Pharmaceutical University Nanjing China; 3 School of Computer Engineering Jinling Institute of Technology Nanjing China; 4 Department of Pharmacy Nanjing Drum Tower Hospital Nanjing China; 5 Department of Clinical Pharmacology Nanjing First Hospital Nanjing Medical University Nanjing China; 6 Laboratory of Drug Informatics Gifu Pharmaceutical University Gifu Japan

**Keywords:** deep learning, BERT, adverse drug reaction, named entity recognition, electronic medical records

## Abstract

**Background:**

With the increasing variety of drugs, the incidence of adverse drug events (ADEs) is increasing year by year. Massive numbers of ADEs are recorded in electronic medical records and adverse drug reaction (ADR) reports, which are important sources of potential ADR information. Meanwhile, it is essential to make latent ADR information automatically available for better postmarketing drug safety reevaluation and pharmacovigilance.

**Objective:**

This study describes how to identify ADR-related information from Chinese ADE reports.

**Methods:**

Our study established an efficient automated tool, named BBC-Radical. BBC-Radical is a model that consists of 3 components: Bidirectional Encoder Representations from Transformers (BERT), bidirectional long short-term memory (bi-LSTM), and conditional random field (CRF). The model identifies ADR-related information from Chinese ADR reports. Token features and radical features of Chinese characters were used to represent the common meaning of a group of words. BERT and Bi-LSTM-CRF were novel models that combined these features to conduct named entity recognition (NER) tasks in the free-text section of 24,890 ADR reports from the Jiangsu Province Adverse Drug Reaction Monitoring Center from 2010 to 2016. Moreover, the man-machine comparison experiment on the ADE records from Drum Tower Hospital was designed to compare the NER performance between the BBC-Radical model and a manual method.

**Results:**

The NER model achieved relatively high performance, with a precision of 96.4%, recall of 96.0%, and F1 score of 96.2%. This indicates that the performance of the BBC-Radical model (precision 87.2%, recall 85.7%, and F1 score 86.4%) is much better than that of the manual method (precision 86.1%, recall 73.8%, and F1 score 79.5%) in the recognition task of each kind of entity.

**Conclusions:**

The proposed model was competitive in extracting ADR-related information from ADE reports, and the results suggest that the application of our method to extract ADR-related information is of great significance in improving the quality of ADR reports and postmarketing drug safety evaluation.

## Introduction

Adverse drug reactions (ADRs) are a significant factor influencing the efficacy and safety of drugs and sometimes may even be life-threatening [1]. These safety problems are recorded as adverse drug events (ADEs) and reported to a special system like the Spontaneous Reporting System, which receives information from a wide range of sources, such as hospitals, small clinics, pharmacies, drug manufacturers, surveillance departments, and individuals [2]. Consequently, collecting and analyzing the ADEs that were recorded in the ADR reports have provided important content for drug safety supervision [3]. Conventional utilization of ADR reports mainly focuses on direct statistical analyses of structured sections [4,5], while the free-text section is quite underutilized because of the unstructured format. The unstructured part mainly describes the occurrence process of ADRs, which is a reference for supervisors to evaluate the potential ADRs. It involves a large amount of manual reading and a judgment process in the review step, which reduces the efficiency of evaluation and increases errors. Therefore, developing an automatic extraction tool to extract unstructured ADR-related information from Chinese ADE records is essential to improve the quality of ADR reports and postmarketing drug safety evaluation.

Named entity recognition (NER) is the main task of information extraction, in addition to natural language processing (NLP), the aim of which is to convert the unstructured contents into structured information. In the field of NLP, Word2Vec and other word vector methods [6-8] have been used for a long time to encode the text, which may bring only limited improvement to subsequent NLP tasks and fail to solve the polysemy problems [9,10]. Recently, numerous pretraining language models [11-13] have been proposed one after another, and Bidirectional Encoder Representations from Transformers (BERT) can greatly improve the performance of domain-related NLP tasks when it is fine-tuned with specific field datasets. BERT for Biomedical Text Mining (BioBERT) [14] was pretrained on large-scale biomedical corpora, which outperforms BERT on biomedical NER tasks, biomedical relation extraction tasks, and biomedical question answering tasks. And clinical NER (CNER) [15] also pretrained the BERT model on a large number of Chinese clinical literature sources crawled from the internet. Considering the background of ADRs in this study, we also collected a dataset of ADRs, and BERT was fine-tuned on this large unlabeled Chinese ADR-related corpus. As for the NER tasks, from the early dictionary-based [16] and rule-based method [17] to the traditional machine learning method [18] and then to the deep learning–based method, bidirectional long short-term memory (bi-LSTM) and conditional random field (CRF) have been widely used in the NER tasks. Wei et al [19] fused the results of CRF with those of bidirectional recurrent neural network (bi-RNN) by support vector machine and finally obtained a higher F1 score than those from CRF or bi-RNN models alone. The hybrid model of LSTM and CRF was proposed by Lample et al [20] in 2016, and its outstanding performance in many NER studies has made it the most popular NER model in recent years.

Consequently, in our study, we created a novel model, BERT-Bi-LSTM-CRF-Radical (BBC-Radical), that took token features and radical features as input and accurately recognized target entities in the sentence with the Bi-LSTM-CRF model. In order to better verify the performance of the model in the real world, we designed a Man-Machine comparison experiment based on the ADEs recorded by the Drum Tower Hospital from 2016 to 2019. We found that our method had excellent performance and efficiency (precision: 87.2%; recall: 85.7%; F1 score: 86.4%) versus manual method (precision: 86.1%; recall: 73.8%; F1 score: 79.5%). The automatically extracted ADR-related entities can further jointly serve as resources for ADR evaluation. On the whole, our study presented a novel method to identify ADR-related information from Chinese ADE reports.

## Methods

### Study Components

**Figure 1:**
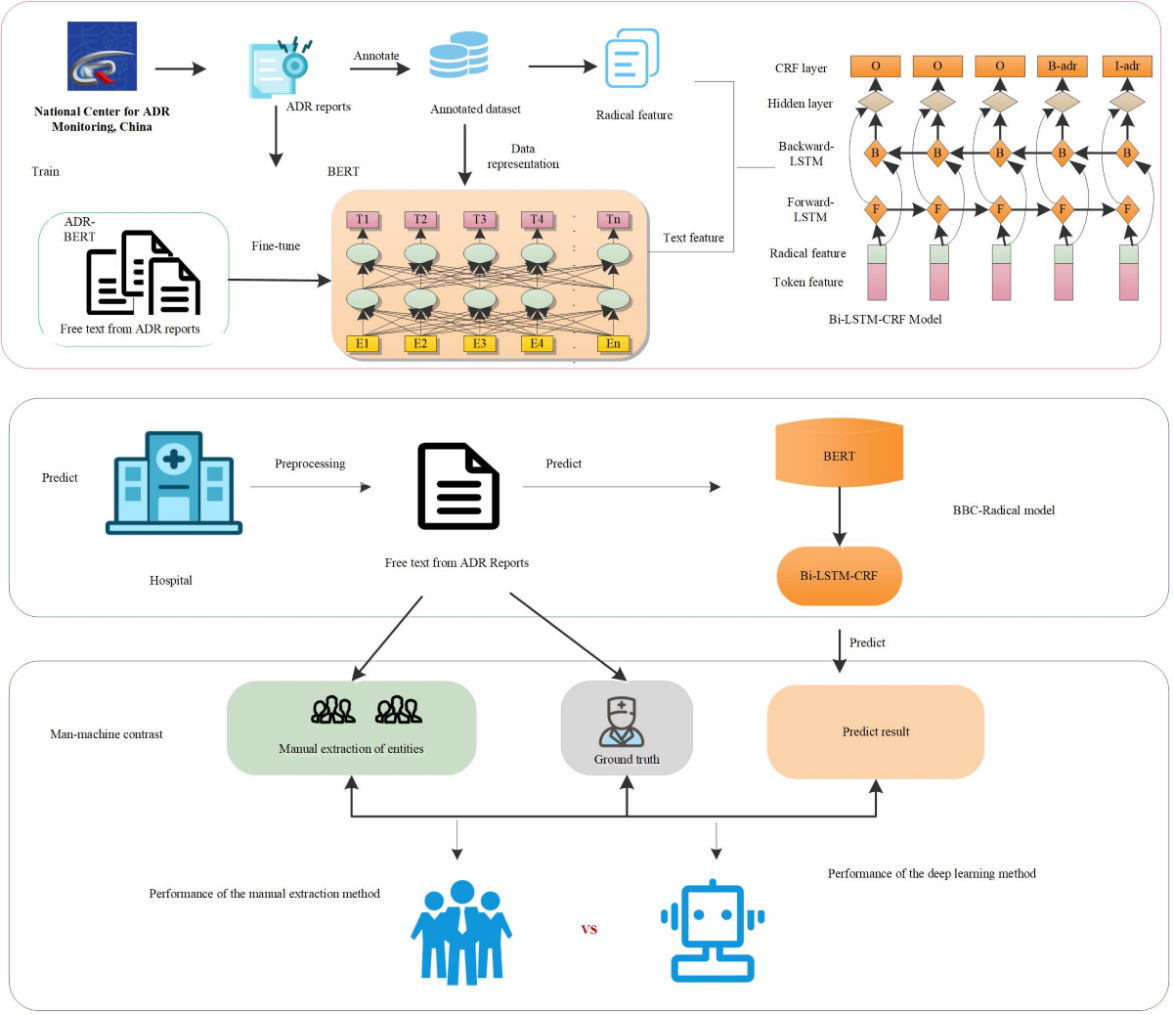
The pipeline in our study; when training a named entity recognition (NER) mode, the data representation model based on the Bidirectional Encoder Representations from Transformers (BERT) model and the combination of token features (pink boxes) and radical features (green boxes) were fed into the bidirectional long short-term memory-conditional random field (bi-LSTM-CRF) model. ADR: adverse drug reaction, BBC-Radical: BERT-Bi-LSTM-CRF-Radical.

In our study, the model conducted the NER task in the free-text section of the Chinese ADE report from 2010-2016 from the Jiangsu province ADR Monitoring Center. According to the original content and the structure characteristics of the ADR cases and combined with other related research corpus annotation process, we established annotation rules and a tool for this study to recognize the entities and entity relationships between parts of the corpus annotation. In addition, the Man-Machine comparison experiment based on the ADE recorded by the Drum Tower Hospital was conducted to verify the extrapolation and robustness of the new data in the model. Figure 1 shows the pipeline of our study. The whole study can be divided into 3 parts: (1) training an NER model. The data representation model based on the BERT and the combination of token features (pink boxes) and radical features (green boxes) were fed into the Bi-LSTM-CRF model. Then, (2) the model performance was verified with real external data, and (3) a Man-Machine comparison experiment was designed to compare the efficiency and accuracy of NER tasks with a manual extraction method and a deep learning method.

### Dataset and Data Annotation

An ADR report can commonly be divided into 2 parts: structured section and free-text section. The data we used in this paper were from the unstructured section of Chinese ADR reports from the ADR monitoring center of Jiangsu Province in 2010-2016. The free-text section of a Chinese ADE report is the narrative content of the ADE procedure, commonly consisting of the process, solutions, and results of ADEs, along with the reasons for the medications being used to generate or degenerate the ADEs, in the form of one or more sentences, which may include some information that has not been recorded in the structured section. In this way, we applied NER technologies to extract entities automatically from these texts, which can be an auxiliary tool for ADR evaluation.

We manually annotated 24,890 cases from the free-text section from Chinese ADR reports, which have been described in [21]. To cover most of the cases, only 3 entities (“Reason,” “Drug,” and “ADR”) were annotated, with some other entities of low frequency not taken into consideration. The annotation rules and examples of entities are shown in Table 1.

**Table 1:** The definition and annotation rules and examples of entity annotation.

Entities and annotation rules		Examples
**Reason^a^**		
	Symptoms or disease states associated with drug use	Diabetes, fever
	Treatment involved with drug use	Postoperative fever
**Drug^b^**		
	Generic names of medications	Levofloxacin
	Trade names of medications	Lipitor
	Abbreviations of medications	10% GS, 0.9% NS
**ADR^a,c^**		
	Adverse reactions during or after medication	Bellyache

^a^Reference for the disease and adverse reaction definitions and classifications is the international Medical Dictionary for Regulatory Activities (MedDRA).

^b^“Drug” entity contains the generic name, trade name, abbreviation, and dosage form adjacent to the drug.

^c^ADR: adverse drug reaction.

#### Input Representation of NER

To improve the efficiency of annotating work, the labeled cases were annotated by an efficient tool [21,22]. We used a special [CLS] at the beginning of the sentence, used [SEP] to separate segments or denote the end of the sequence, and added [PAD] tokens at the end of the sentences to make their lengths equal to the maximum sequence length. Finally, 24,890 valid annotated cases were obtained, including 147,451 entities in the Chinese ADR reports.

#### BBC-Radical Method

**Figure 2:**
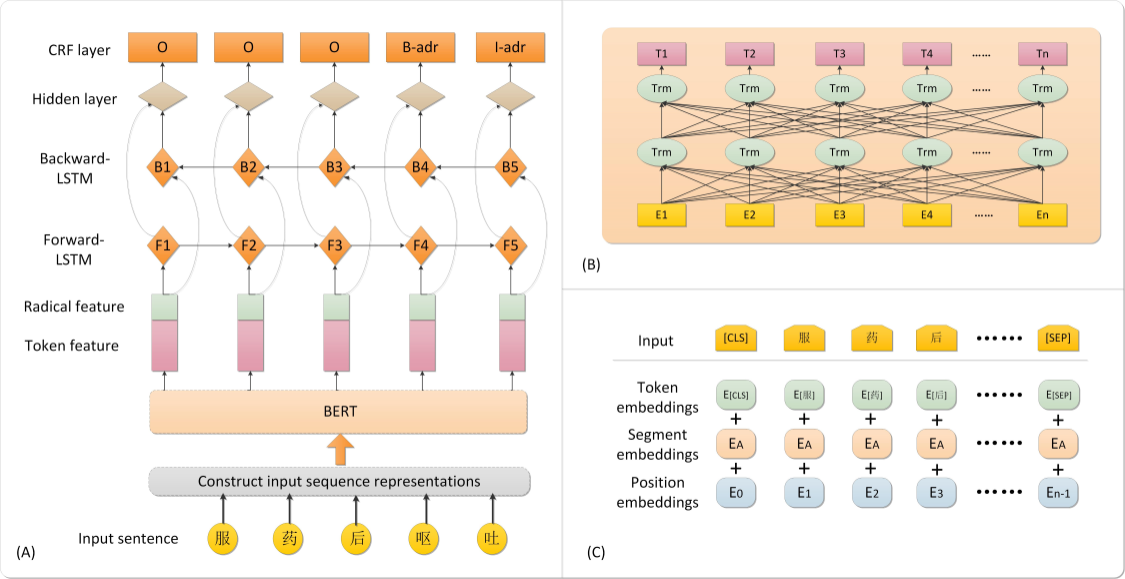
(A) Architecture diagram of our proposed model, in which the combination of token features (pink boxes) and radical features (green boxes) were fed into the bidirectional long short-term memory-conditional random field (Bi-LSTM-CRF) model; (B) data representation model based on the Bidirectional Encoder Representations from Transformers (BERT) model, in which the sequence of [E_1, E_2, E_3 … E_n] in the yellow boxes is the input to the BERT model and the green ellipses represent the Transform blocks; and (C) construction of input sequence representations for the BERT, in which the input is composed of token embedding, segment embedding, and position embedding.

Figure 2B shows the data representation model based on BERT that takes the corresponding token, segment, and position embeddings of each word as inputs in Figure 2C. The contextual embeddings for each token can be obtained from the output of the BERT model, which is the token feature input of the next NLP task.

Owing to the fact that the corpus we used in this study is highly domain-specific, we also collected nearly 461,930 ADE records from the ADR Monitoring Center of Jiangsu Province ranging from 2010 to 2016 to improve the precision of domain-specific word representation. These records were mainly from medical personnel in hospitals and pharmacies and from follow-up records from pharmaceutical companies. The diversity of submitting agencies and reporters enriched the sample and made the language characteristics of the data sources more complex. To fine-tune BERT, we first generated a pretraining data (a tfrecord file) file with the clinical text. Then, we pretrained our fine-tuned BERT model on the pretraining file from the existing BERT checkpoint of the original language model (BERT_BASE-Chinese-uncased_). Once the fine-tuning process was completed, we got a TensorFlow model that was transformed to a PyTorch model for further NER tasks.

Radical is a common form extracted from many Chinese characters, so that these characters not only have the basis of classification in the form but also become a common genus in the meaning of the word, which is helpful for people to summarize the meaning of the word. Therefore, the meaning of radicals is very important for people to grasp the meaning of a word. Moreover, the radical features of Chinese characters have been widely used to enhance different Chinese NLP tasks in recent years [23-25]; consequently, in addition to considering Chinese characters themselves, we also considered applying radical features to the model. The overall network architecture of our NER model is shown in the Bi-LSTM-CRF in Figure 2A. In our study, each token in our sequence was fed into the fine-tuned BERT model to train for the data representation of the whole sequence. After obtaining a representation of the entire sequence [*T*_1_, *T*_2_, *T*_3_, …*T*_n_], we looked for the radical of each word in the sequence and initialized each radical with random values to indicate the radical feature. The concatenation *x* = [*w*_1, *w*_2, *w*_3, … *w*_4] of the word vectors and the radical vectors were fed into the Bi-LSTM model, and the context vectors learned by forward and backward LSTM layers were then transmitted into the CRF layer to compute the corresponding probability values and to simultaneously predict tags. The details of our NER method in the Bi-LSTM-CRF are shown in Multimedia Appendix 1. We also implemented 3 baselines for the NER task, as follows:

1. CRF + + is a well-known open-source tool for CRF that is also the CRF tool with the best comprehensive performance at present.
2. The Bi-LSTM-CRF model that takes the input representation trained by Word2Vec as input was used as a baseline.
3. The combined model, BBC-Radical model without fine-tuning BERT on domain-specific corpus, was also used as a baseline (BERT + Bi-LSTM-CRF-Radical). The model architectures and experimental settings were the same as in our proposed model.

## Results

### Experimental Settings

All the models were trained on an NVIDIA Tesla V100 GPU with 768 GB of memory using the PyTorch framework. The longest length of a sentence can be set to 512 in the fine-tuned BERT of our NER model. To maintain the complete information from the sentences, the excess part was split into another sentence once the length exceeded 512 tokens, until all the segmented sentences could satisfy the length constraint. We trained the model with a batch size of 16, the hidden unit of bi-LSTM was 128, and we also used radical embedding, which was initialized with 20 random values. We also set the initial learning rate as 510^-5^ in the Adam optimizer.

### Evaluation Metrics

The results were measured using a micro-averaged F1 score = (2*PR*)/(*P* + *R*), where *P* denotes precision and *R* represents recall. In our research, we followed the strict matching rule that was defined at the start and end boundaries, and the extraction result referred to the same entity types as the ground truth.

### Findings

In the NER task, 15,000 and 8000 cases were randomly selected separately from the annotated cases as the training set and testing set, respectively, and the remaining 1890 cases were considered the validation set, which was used to verify the generalization ability of the model in the training process. In order to better evaluate the model, we ran our proposed model and the third baseline model 10 times, keeping all the other parameters the same except the sampled training data. The average value of each valuation metric was used to show the prediction results in Table 2.

**Table 2:** Overall concept extraction performances from the free-text section of Chinese adverse drug reaction (ADR) reports.

Model	Precision (%), mean (SD)	Recall (%), mean (SD)	F1 score (%), mean (SD)
CRF^a^ ++ [21]	94.4 (0.32)	93.1 (0.28)	93.9 (0.08)
Word2Vec + Bi-LSTM^b^-CRF [21]	94.6 (0.33)	94.1 (0.30)	94.4 (0.29)
BERT^c^ + Bi-LSTM-CRF-Radical	95.2 (0.07)	95.2 (0.07)	95.2 (0.06)
Fine-tuning BERT + Bi-LSTM-CRF	96.0 (0.05)	95.5 (0.08)	96.0 (0.06)
Fine-tuning BBC^d^-Radical	96.4 (0.04)	96.0 (0.03)	96.2 (0.04)

^a^CRF: conditional random field.

^b^Bi-LSTM: bidirectional long short-term memory.

^c^BERT: Bidirectional Encoder Representations from Transformers.

^d^BBC: BERT-Bi-LSTM-CRF.

CRF is a probabilistic structure model by marking and segmenting sequence data. The obvious disadvantage of the word vector model represented by Word2vec is that it is context-free, which results in the same word having the same meaning in different contexts. Consequently, neither the CRF++ nor the second baseline model did very well in our NER task. The third row in Table 2 represents the model combining the original BERT and Bi-LSTM-CRF-Radical models, and the BERT model in our proposed BBC-Radical model was fine-tuned on the domain-specific corpus. The results in the fourth row of Table 2 also show the contribution of radical embedding in the NER task. The proposed model in our research achieved an F1 score of 96.2%, which outperforms the 4 baseline models. The results showed that BERT plays an important role in capturing more text information, and our pre-trained BERT on a specific domain can significantly improve the performance of entity extraction.

**Figure 3:**
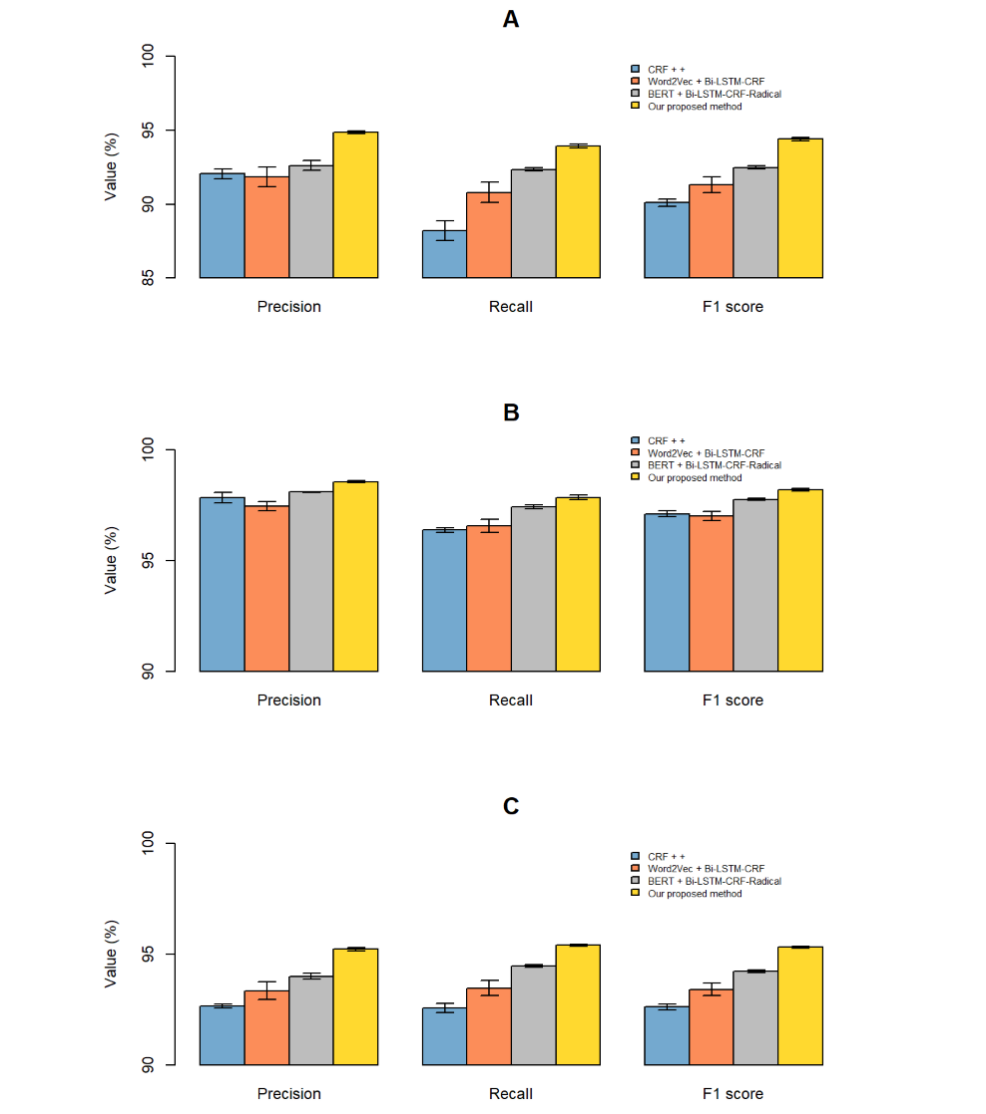
Precision, recall, and F1 score for each kind of entity: (A) reason, (B) drug, and (C) adverse drug reaction (ADR). CRF: conditional random field; BERT: Bidirectional Encoder Representations from Transformers; bi-LSTM: bidirectional long short-term memory.

Our proposed method outperformed the other methods for all entity types, and the entity of “Drug” achieved the highest F1 score, while the entity of “Reason” achieved the lowest (Figure 3). This can be implied from the definitions of each entity, in which the entity of “Reason” included not only conventional diseases and symptoms but also some other treatments involved with drug use, along with their body parts and adjoining adjectives, while the definitions of “Drug” and “ADR” were relatively simpler. Because the definitions or annotations of the rules were more varied, the error rate of the model was relatively high. The overlap of the concepts of different kinds of entities is another reason for the false recognition between “Reason” and “ADR.” For instance, the entity of “Reason” was always recorded in ADR reports with the colloquial expressions of symptoms. And it was hard to recognize the “Reason” of “anorexia” when it was recorded as “never feel like eating.” As for the entity of “Drug,” we found that it was difficult to identify infrequently used trade names, some English abbreviations, and some traditional Chinese medicines that are composed of peculiar characters.

### Validation Results

#### The Man-Machine Contrast on the External Validation Dataset

As the frontier direction of artificial intelligence research, man-machine confrontation technology has always been a hot spot of artificial intelligence research. Research of artificial intelligence, mainly in the form of man-machine confrontation, provides an excellent experimental environment and verification method for exploring the internal growth mechanism and key technical principles of machine intelligence. The whole process can not only make the machine serve humans more intelligently but also free humans from some complex tasks. For further validation, we selected 2479 ADE reports recorded by physicians from The Drum Tower Hospital, School of Medicine, Nanjing University from 2016 to 2019 and validated our proposed model to conduct NER experiments on the descriptive texts of adverse events. After professional training, the ground truth for the 2479 cases was produced by 10 students majoring in hospital pharmacy, who spent 2 weeks for 4 rounds of annotating (including 1 round of pre-annotating, 2 rounds of formal annotating, and 1 round of annotation correction). The rules for annotation were the same as those for our annotation training set. In order to further illustrate the advantages of the entity recognition model in this paper, we designed a Man-Machine comparison experiment. In reality, the hospital reports the ADEs to the ADR center every year, and then, the staff of the adverse reaction center needs to review and return reports that do not conform to the standard. Therefore, there are some differences in the recognition performance between the ADEs reported by the hospital and the data from the ADR monitoring center. As for the manual method, we invited 5 additional pharmacy students to participate in the experiment. Under the guidance of the ADR supervisor, the 5 students were required to complete the entity extraction of the assigned data by manual search within 2 weeks after training. Since manual entity extraction is time-consuming and laborious, we only had 2 rounds of marking, and finally, we obtained the results of manual entity extraction. The results of the Man-Machine comparison to the external validation data are shown in Multimedia Appendix 2.

#### Comparison of the Man-Machine Contrast Results

**Figure 4:**
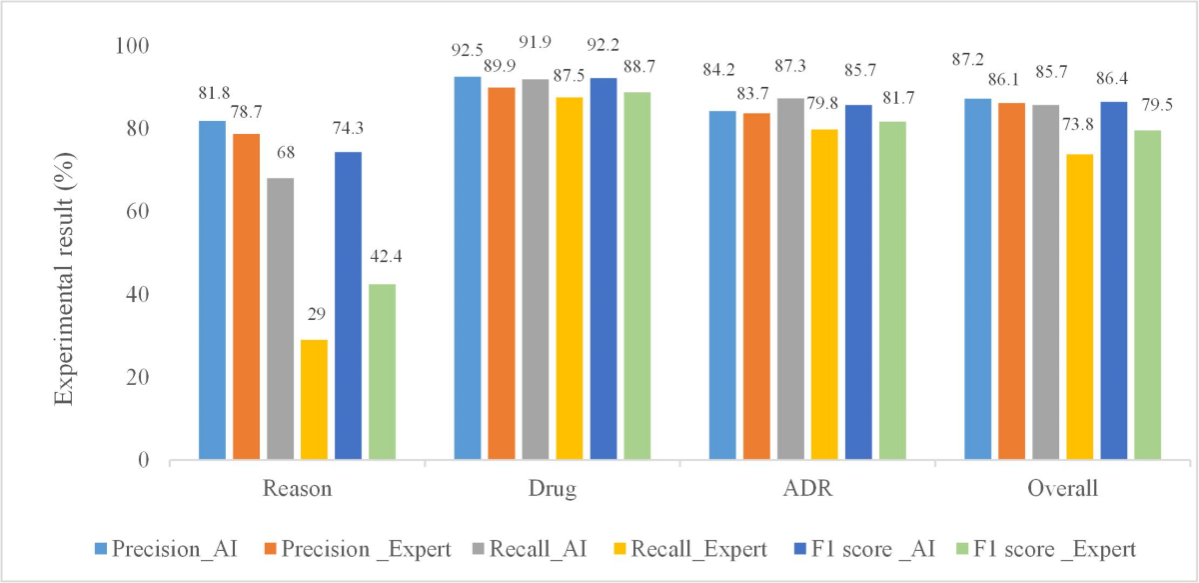
Comparison of the Man-Machine contrast. ADR: adverse drug reaction.

After the preprocessing step, the validation data were fed into our model for prediction, and the performance of the prediction results is provided in Figure 4 (light blue, gray, and blue bars). From the perspective of the entity category, when the target entity “Reason” was only defined as “Disease,” the identification accuracy of the entity was relatively good [26], while our definition of “Reason” also contained other drug use–related treatments and symptoms. Tao et al [27] performed the NER task of “Reason” and other medicine-related entities, and the resulting F1 score for “Reason” was only 40.9%; our F1 score for “Reason” was 74.3%. Figure 4 (orange, yellow, and green bars) also shows the results of the manual extraction method. The manual method achieved a precision of 86.1%, recall of 73.8%, and F1 score of 79.5% for the NER task. Therefore, manual extraction of entities was not only inefficient, but also had low accuracy, especially the identification of the entity “Reason.” The accuracy of “Drug” entity extraction was relatively high due to the normative name of the “Drug” entity in each experiment, while the extraction of the “Reason” and “ADR” entities was also negatively affected by nonstandard documentation.

In Figure 4, the F1 score is only 42.4%, and recall is extremely low in the recognition of “Reason,” far lower than with our deep learning method. A possible reason could be that many entities were not recognized when the entity of “Reason” was identified manually because of limited human attention and accuracy. At the same time, by comparison, we found that the F1 score of the BBC-Radical model for the recognition of other entities was also much better than the manual recognition method. As a special machine learning method, deep learning can automatically extract features from data samples, which reduces the process of constructing artificial features and has more advantages for processing large data sets.

## Discussion

### Principal Findings

In our study, we developed a domain-specific NER method on Chinese ADE records. The extraction of biomedical entities and their relationships from texts is of great application value to biomedical research. Accurately extracting entity information from free text in Chinese ADE reports with NER methods in daily practical work can greatly simplify the approval work of staff in the ADR monitoring center and improve the quality of ADE reports. In addition to using medical reports for detecting ADRs, it has been proposed to use data from social media [28], since users tend to discuss their illnesses, treatments, and prescribed medications and their effects on social media platforms. For example, when Cocos et al [29] and Xie et al [30] extracted ADR entities from social media using dictionary matching, using a CRF and bi-LSTM model, respectively, they helped reduce the limitations of passive reporting systems. Also, the automatic detection of chemical references in biomedical literature is an essential step for further biomedical text mining and has recently received considerable attention. In addition to using a single model for training, Zeng et al [31] and Luo et al [32] achieved high F1 scores when they integrated bi-LSTM and CRF to extract the drug entity and chemical substance entity, respectively, from the text. The performance of the baseline model using a single CRF + + also proved that the single CRF model was inferior to the BI-LSTM-CRF model. Due to the excellent performance of the hybrid model with bi-LSTM and CRF, the hybrid model architecture with bi-LSTM and CRF was also applied in the entity extraction layer of our proposed model.

Most NLP tasks based on deep learning can be divided into the following 3 modules: data processing, text representation, and a task-specific model. Word2Vec, GloVe, and BERT are excellent models of text representation that are widely used in different NER models. Chen et al [21] obtained a high F1 score when Word2Vec and Bi-LSTM-CRF were used to extract the named entities from the free-text section of Chinese ADR Reports. However, Chen et al [21] applied Word2Vec in the input layer to generate the data representation, which would bring only limited improvement to subsequent NLP tasks and failed to solve the polysemy problem. In Chinese text, Zhang et al [33] extracted breast cancer–related entities with a pretrained BERT model that was trained on a large-scale, unlabeled corpus of Chinese clinical text. However, their pretrained BERT on this domain was aimed at breast cancer, not general medical records. In our study, we also established a deep neural network algorithm based on a domain-specific BERT model, and our model proved the competitive performance of NER on ADE text in the setting of the same training set with a higher F1 score. From the results of the Man-Machine comparison experiment, our proposed method achieved a high degree of agreement with ground truth. Moreover, the method proposed in this paper was superior to manual extraction in the accuracy and speed of NER.

Furthermore, the use of NER in NLP technology can achieve the target entity in automatic extraction from free text, and the extraction of information can be further used in statistical analysis, such as knowledge base–building tasks. Besides that, the model can be used to automatically extract ADR-related information from electronic medical records or other relevant texts to further supplement the information contained in ADR reports.

### Conclusion

In this study, we explored an NER task on Chinese ADR reports, with an optimized deep learning method of BBC-Radical, which took radical features of each token, token features obtained from the fine-tuned BERT model as the input, and Bi-LSTM-CRF as the feature extract model. The performance of our model was compared with other baseline models on the same dataset, and the experimental results indicated that the BBC-Radical model outperformed other models and obtained a competitive F1 score of 96.2%. Moreover, in the Man-Machine comparison experiment, our method had an absolute advantage over the manual extraction method in terms of time, efficiency, and accuracy. This study conducted a domain-specific NER task in Chinese ADE records, which may play a role in promoting ADR evaluation and postmarketing evaluation of drug safety.

## Appendix

Multimedia Appendix 1 Details of our named entity recognition method.

Multimedia Appendix 2 Results from the external validation data.

## Abbreviations

ADE: adverse drug event
ADR: adverse drug reaction
BBC-Radical: BERT-Bi-LSTM-CRF-Radical
BERT: Bidirectional Encoder Representations from Transformers
bi-LSTM: bidirectional long short-term memory
bi-RNN: bidirectional recurrent neural network
BioBERT: BERT for Biomedical Text Mining
CNER: clinical NER
CRF: conditional random field
NER: named entity recognition
NLP: natural language processing
