# Supplementary material for: Chinese-Named Entity Recognition From Adverse Drug Event Records: Radical Embedding-Combined Dynamic Embedding–Based BERT in a Bidirectional Long Short-term Conditional Random Field (Bi-LSTM-CRF) Model
Source: JMIR Med Inform. 2021 Dec 1;9(12):e26407. doi: 10.2196/26407 (PMC8686410; doi:10.2196/26407)
Supplement: Multimedia Appendix 1 [file medinform_v9i12e26407_app1.docx]

Details of our NER method

The concatenation$x$= [$w_{1}$, $w_{2}$, $w_{3}$ … $w_{n}$] of the word vectors and the radical vectors were feed into the Bi-LSTM model and the context vectors learned by forward and backward LSTM layers were then transmitted into the CRF layer to compute the corresponding probability values and at the same time predict tags.

$f_{t}=\sigma\left( W_{f}x_{t}+b_{f}+U_{f}h_{t-1}+b_{hf} \right)$ (1)

$i_{t}=\sigma\left( W_{i}x_{t}+b_{i}+U_{i}h_{t-1}+b_{hi} \right)$ (2)

$C_{t}=f_{t}C_{t-1}+i_{t}*(tanh (W_{C}x_{t}+b_{c}+U_{C}h_{t-1}+b_{hc}))$ (3)

$O_{t}=\sigma(W_{o}x_{t}+b_{o}+U_{o}h_{t-1}+b_{ho})$ (4)

$h_{t}= O_{t}* tanh(C_{t})$ (5)

In LSTM, related values at the $t$th hidden layer can be calculated as equation (1)~(5), in which $\sigma$ denotes the activation function, $W$ and $U$ denote weights, $b$ denotes bias, $C_{t}$ denotes cell memory, and $h_{t}$ is the final output. In Bi-LSTM, $\vec{h}_{t}$ and $\overleftarrow{h}_{t}$ are used to denote the forward and backward output. And the CRF scores can be calculated as:

$P_{t}=softmax(W_{l}h_{t}+b_{l})$ (6)

where $P_{t}$ denotes the probability of word $x_{t}$ tags to the label $y_{ti}$, $W$ and $b$ denote weights and bias. And $s_{(X,y)}$ is the score of the label sequence $y$ of input sentence, in which $T$ denotes the transition matrix between labels $y_{t}$ and $y_{t+1}$.

$s_{(X,y)}=\sum_{t=0}^{n-1} T_{y_{t,}y_{t+1,}}+\sum_{t=0}^{n} P_{t}$ (7)

The probability of the occurrence of the prediction sequence $y$ is:

$P_{(y|X)}=\frac{e^{s_{(X,y)}}}{\sum_{z\in Y_{X}} e^{s_{(X,z)}}}$ (8)

And $z$ denotes as all the possible label for a sentence. The likelihood function of the prediction sequence is obtained by taking the logarithm of both ends:

$\log P_{(y|X)}=s_{(X,y)}-log(\sum_{z} e^{s_{(X,z)}})$ (9)

$Y={argmax}_{(z\in Y_{X})}( s_{\left( X,z \right)})$ (10)

Finally, we obtained the highest score $Y$ as predicted label.
