# Supplementary material for: Chinese-Named Entity Recognition From Adverse Drug Event Records: Radical Embedding-Combined Dynamic Embedding–Based BERT in a Bidirectional Long Short-term Conditional Random Field (Bi-LSTM-CRF) Model
Source: JMIR Med Inform. 2021 Dec 1;9(12):e26407. doi: 10.2196/26407 (PMC8686410; doi:10.2196/26407)
Supplement: Multimedia Appendix 2 [file medinform_v9i12e26407_app2.docx]

Results from the external validation data

To further evaluate the performance of entity recognition model in this paper, we designed a Man-Machine comparison experiment. Table S1 shows the results of the prediction results on our NER model with the rows representing the experimental metrics of different kind of entity type.

Table S1. The Precision, Recall and F1 score each kind of entity and corresponding overall results of our NER model on external validation data.

| Entity | Precision (%) | Recall (%) | F1 (%) |
| --- | --- | --- | --- |
| Reason | 81.79 | 67.98 | 74.25 |
| Drug | 92.45 | 91.88 | 92.16 |
| ADR | 84.19 | 87.29 | 85.71 |
| Overall | 87.17 | 85.69 | 86.43 |

Comparing manual and automated extraction of ADR associated entities from ADR reports, the manual method is time-consuming and laborious, resulting in error-prone inefficiency. Table S2 indicated the precision, Recall and F1 score of each entity and corresponding overall results of manual extraction. It indicated that there are still lots of cases which are wrongly classified from the result of Man-Machine contrast. The Recall is a measure of coverage, which indicates how many positive examples in the sample are predicted correctly. It is found that most positive samples are wrongly recognized as negative samples when the Recall was low, so the ability of the model to learn positive examples becomes weaker. In our manual method, many entities were not recognized when the entity of “Reason” was identified manually, resulting in the Recall only 29.03% for entity recognition of “Reason”, which due to the limited time and energy of manual recognized. Staring at a computer screen for long periods of time can blur your vision and dissipate your energy, which can lead to mismarks or missing annotates.

Table S2. The Precision, Recall and F1 score of each kind of entity and corresponding overall results on external validation data of manual extraction.

| Entity | Precision (%) | Recall (%) | F1 (%) |
| --- | --- | --- | --- |
| Reason | 78.70 | 29.03 | 42.42 |
| Drug | 89.92 | 87.48 | 88.68 |
| ADR | 83.69 | 79.76 | 81.68 |
| Overall | 86.13 | 73.80 | 79.49 |
